# Comparison of the methods for isolation and detection of SARS-CoV-2 RNA in municipal wastewater

**DOI:** 10.3389/fpubh.2023.1116636

**Published:** 2023-03-07

**Authors:** Vincent Lucansky, Marek Samec, Tatiana Burjanivova, Eva Lukacova, Zuzana Kolkova, Veronika Holubekova, Eva Turyova, Andrea Hornakova, Tibor Zaborsky, Petar Podlesniy, Lenka Reizigova, Zuzana Dankova, Elena Novakova, Renata Pecova, Andrea Calkovska, Erika Halasova

**Affiliations:** ^1^Biomedical Centre Martin, Jessenius Faculty of Medicine in Martin (JFMED CU), Comenius University in Bratislava, Martin, Slovakia; ^2^Department of Pathophysiology, Jessenius Faculty of Medicine in Martin, Comenius University in Bratislava, Martin, Slovakia; ^3^Department of Molecular Biology and Genomics, Jessenius Faculty of Medicine in Martin, Comenius University in Bratislava, Martin, Slovakia; ^4^RÚVZ (Regional Office of Public Health), Martin, Slovakia; ^5^Centro Investigacion Biomedica en Red Enfermedades Neurodegenerativas (CiberNed), Madrid, Spain; ^6^Center for Microbiology and Infection Prevention, Department of Laboratory Medicine, Faculty of Health Care and Social Work, Trnava University, Trnava, Slovakia; ^7^Biobank for Cancer and Rare Diseases, Jessenius Faculty of Medicine in Martin (JFMED CU), Comenius University in Bratislava, Martin, Slovakia; ^8^Department of Microbiology and Immunology, Jessenius Faculty of Medicine in Martin, Comenius University in Bratislava, Martin, Slovakia; ^9^Department of Physiology, Jessenius Faculty of Medicine in Martin, Comenius University in Bratislava, Martin, Slovakia

**Keywords:** SARS-CoV-2, wastewater, WBE, droplet digital PCR (ddPCR), real-time quantitative polymerase chain reaction (RT-qPCR), COVID-19

## Abstract

**Introduction:**

Coronavirus SARS-CoV-2 is a causative agent responsible for the current global pandemic situation known as COVID-19. Clinical manifestations of COVID-19 include a wide range of symptoms from mild (i.e., cough, fever, dyspnea) to severe pneumonia-like respiratory symptoms. SARS-CoV-2 has been demonstrated to be detectable in the stool of COVID-19 patients. Waste-based epidemiology (WBE) has been shown as a promising approach for early detection and monitoring of SARS-CoV-2 in the local population performed *via* collection, isolation, and detection of viral pathogens from environmental sources.

**Methods:**

In order to select the optimal protocol for monitoring the COVID-19 epidemiological situation in region Turiec, Slovakia, we (1) compared methods for SARS-CoV-2 separation and isolation, including virus precipitation by polyethylene glycol (PEG), virus purification *via* ultrafiltration (Vivaspin^®^) and subsequent isolation by NucleoSpin RNA Virus kit (Macherey-Nagel), and direct isolation from wastewater (Zymo Environ Water RNA Kit); (2) evaluated the impact of water freezing on SARS- CoV-2 separation, isolation, and detection; (3) evaluated the role of wastewater filtration on virus stability; and (4) determined appropriate methods including reverse transcription-droplet digital PCR (RT-ddPCR) and real-time quantitative polymerase chain reaction (RT-qPCR) (targeting the same genes, i.e., *RdRp* and gene *E*) for quantitative detection of SARS-CoV-2 in wastewater samples.

**Results:**

(1) Usage of Zymo Environ Water RNA Kit provided superior quality of isolated RNA in comparison with both ultracentrifugation and PEG precipitation. (2) Freezing of wastewater samples significantly reduces the RNA yield. (3) Filtering is counterproductive when Zymo Environ Water RNA Kit is used. (4) According to the specificity and sensitivity, the RT-ddPCR outperforms RT-qPCR.

**Discussion:**

The results of our study suggest that WBE is a valuable early warning alert and represents a non-invasive approach to monitor viral pathogens, thus protects public health on a regional and national level. In addition, we have shown that the sensitivity of testing the samples with a nearer detection limit can be improved by selecting the appropriate combination of enrichment, isolation, and detection methods.

## 1. Introduction

SARS-CoV-2 is a novel member of the coronavirus genus identified in late 2019. It is a causative agent of the infectious disease COVID-19 that can lead to a wide range of manifestations from mild respiratory symptoms or even an asymptomatic course to severe viral pneumonia resulting in death (1). Due to high infectivity and death toll, COVID-19 has become a disease with a significant impact on the health status of the world population as well as on the world economy and politics. On January 30, 2020, the World Health Organization (WHO) Emergency Committee designated COVID-19 as a global health emergency (2). Approximately 648,131,832 cases and 6,640,702 deaths have been attributed to COVID-19 worldwide so far (December 1^st^, 2022) (3).

Testing soon became an essential part of the COVID-19 pandemic management. However, the strategical selection of appropriate diagnostic approach and its settings is crucial for the quality and usefulness of the COVID-19 testing. An attempt to perform nationwide screening (4) turned out to be cost-ineffective, unpopular, logistically challenging, and potentially risky due to the huge spatiotemporal accumulation of tested individuals. Moreover, the nationwide screening did not provided any long-term improvement of the epidemiological situation or gave any reliable information about the actual dynamics of the pandemic. As we showed in our previous work (5), the usage of rapid antigen testing is not reliable due to a high number of misdiagnosed false-negative virus carriers. The individual real-time quantitative polymerase chain reaction (RT-qPCR) testing is also biased due to the fact that many tested subjects undergo diagnostic procedures not randomly but their participation is motivated by symptom occurrence either on themselves or on the person sharing their work or living environment, thus driven by suspicion. Vice versa, many individuals with in apparent infection are not tested and are not included in case reports.

On the contrary, environmental monitoring of wastewater is independent of the testing of individuals and can therefore become a critical tool for monitoring the epidemiological situation of COVID-19 (6, 7). The presence of SARS-CoV-2 in wastewater has been reported in several studies (6–10). Based on clinically confirmed cases, the observed viral titers were significantly higher than expected viral titers (11). The correlation between SARS-CoV-2 RNA concentration in sewer and the occurrence of new cases has showed to be stronger than that of active cases and cumulative cases obtained by individual testing (12).

Hence, we decided to test several protocols enabling COVID-19 surveillance in sewer water. In particular, we established three concentration protocols for the enrichment of viral RNA from wastewater and its subsequent isolation, followed by two different detection methods. We are aware that the spectrum of techniques is much broader. However, our selection was influenced by a combination of our previous hands-on experience, workplace availability as well as an effort to cover principally different approaches. For the enrichment of viral particles in wastewater, polyethylene glycol (PEG) precipitation and centrifugal membrane concentrator protocols were used. Both methods were followed by an utilization of virus RNA isolation kit. The third approach was applied using the kit combining viral enrichment with RNA isolation in a single protocol. Subsequent detection was performed with the utilization of either quantitative RT-PCR (RT-qPCR), the commonly used diagnostic method for SARS-CoV-2 during the COVID-19 pandemic (13), or reverse transcription-droplet digital PCR (RT-ddPCR) that is considered to provide higher sensitivity and specificity rate compared to RT-qPCR RT-ddPCR thus avoid more false-negative results of samples with low viral load (14). Both PCR assays detected matching genes, namely *RdRp* and *E*, which facilitated the comparison of obtained data.

## 2. Materials and methods

### 2.1. Enrichment and SARS-CoV-2 RNA isolation

The wastewater samples were obtained at the sewage treatment plant in Vrútky, which collects wastewater from Martin city and parts of the Turiec region. Sewage water samples for our experiment were collected from April to July 2022. At that time, the epidemiological situation in the Slovak republic was relatively stabilized, characterized by a decline of newly diagnosed cases; thus, SARS-CoV-2 presence in sewage was expected to be at very low levels (see Supplementary Table A in Supplementary material). Samples were transported to the laboratory on ice and then kept at 4°C until further processing on the same day. The first step was debris removal by centrifugation at 4,000 g for 30 min at 4°C. Next, half of the supernatant was filtered using 0.45 μm syringe filters. Samples of both filtered and unfiltered wastewater were either frozen at −20°C or processed further immediately.

For PEG precipitation, the supernatant was incubated with 8% PEG-8000 (Merck), and 0.3 M NaCl (Sigma-Aldrich) overnight (app. for 16 h) at 4°C. Centrifugation was performed at 10,000 g for 120 min, at 4°C, then the supernatant was removed and the pellet was diluted in 500 μl Opti-MEM™ (Gibco).

When using the Vivaspin centrifugal filter device/molecular weight cut-off 50 kDa (Sartorius), the supernatant was concentrated by centrifuging at 4,000 g for 30 min at 4°C. This centrifugal step was repeated to pass through the entire 50 ml supernatant volume until the final volume of the concentrated sample reached 500 μl. Then the enriched sample was collected and further processed.

Further, after both PEG precipitation and centrifugal filter enrichment protocols, total RNA isolation was carried out using the NucleoSpin™ RNA Virus column (Macherey Nagel). Then, RNA was eluted with 30 μl of RNase-free water according to the manufacturer's protocol. All RNA isolations were performed in triplets.

Zymo Environ Water RNA Extraction kit (Zymo) covers viral enrichment, sample homogenization, and RNA purification in one workflow protocol. We used 5 ml of wastewater that was aliquoted into five separated tubes (1 ml/each). Subsequently, we added 70 μl of Water Concentrating Buffer into each tube. Further steps were processed according to the manufacturers' recommendations.

### 2.2. SARS-CoV-2 RNA detection

Detection of SARS-CoV-2 viral RNA in the sample of wastewater was performed by a one-step RT-qPCR method using IVD-certificated kit gb SARS-CoV-2 Multiplex (GENERI BIOTECH s.r.o., Hradec Králové, Czech Republic). This kit allows the detection of viral *E* and *RdRP* genes within one reaction with a limit of detection of 2.13 copies of viral RNA per reaction (95 % CI). To avoid false negative results, the PCR process was verified by external positive control (EPC) added to the reaction. Reactions were prepared according to the manufacturer's instructions. Briefly, the PCR reaction with a volume of 20 μl contained 10 μl of Master Mix OneStep Multi, 5 μl of multiplexed Assay CoV-2 *E*-*RdRP*, 0.25 μl of EPC Template RNA, and 5 μl of extracted RNA. Positive control as well as NTC with distilled water was included in each run. PCR conditions were as follows: reverse transcription at 42°C for 30 min, initial denaturation at 95°C for 3 min, 50 cycles consisting of two steps (denaturation at 95°C for 10 s and annealing plus elongation at 60°C for the 30 s). The fluorescence signal was measured in the FAM channel for viral gene *E*, in the HEX channel for viral *RdRP* gene, and in the Cy5 channel for EPC. Reactions were evaluated as invalid if the signal in the Cy5 channel was not detected.

Reverse transcription-droplet digital PCR (RT-ddPCR) was performed in 20 μl reaction volume, consisting of 17 μl of master mix and 3 μl of the sample. Mastermix contained 5 μl of supermix, 2 μl of reverse transcriptase (RT), and 1 μl of 300 mM dithiothreitol (DTT) solution, all included in One-Step RT-ddPCR Advanced Kit for Probes (Bio-Rad Laboratories, Hercules, California, USA), primers and probes (Generi Biotech, Hradec Králové, Czech Republic) at a final concentration of 500 and 250 nM, respectively. Primer and probe sequences were as follows: RdRp (F): GTGAAATGGTCATGTGTGGCG, RdRp (R): AATGTTAAAAACACTATTAGCATAAGCA, RdRp: CAGGTGGAACCTCATCAGGAGATGC/HEX-IBFQ; E (F): ACAGGTACGTTAATAGTTAATAGCGT, E (R): ATATTGCAGCAGTACGCACA, E: ACACTAGCCATCCTTACTGCGCTTCG/FAM-IBFQ; GAPDH (F): AGTCAGCCGCATCTTCTTTT, GAPDH (R): CCCAATACGACCAAATCCGT, GAPDH: GCGTCGCCAGCCGAGCCACA/HEX-IBFQ. Commercially available SARS-CoV-2 Standard (Exact Diagnostics, Bio-Rad Laboratories, Fort Worth, Texas, USA) manufactured with synthetic RNA transcripts containing five gene targets (*E, N, ORF1ab, RdRP*, and *S* of SARS-CoV-2) was used as a ddPCR quantitative positive control. All samples were analyzed in duplicates for *GAPDH* and single wells for each viral gene, *RdRp* + *GAPDH* and *E* + *GAPDH*. Droplets were generated by an automated droplet generator (Bio-Rad Laboratories, Hercules, California, USA) according to the manufacturer's instructions. PCR was performed using a T100 thermal cycler (Bio-Rad Laboratories, Hercules, California, USA) with the following cycling conditions: reverse transcription at 50°C for 60 min, denaturation at 95°C for 10 min, followed by 40 cycles of denaturation at 94°C for 30 s, followed by annealing/extension at 54°C for 1 min, and droplet stabilization at 98°C for 10 min. Samples were then analyzed using QX200 Droplet Reader (Bio-Rad Laboratories, Hercules, California, USA). Thresholding was carried out by using QuantaSoft Software manually at the lowest amplitude that captures true negative clusters based on the signals of the negative control and positive control samples. The results were reported as positive when at least five copies of each viral gene (*RdRp, E*) occurred (15). Data are interpreted as copies per reaction according to previous works evaluating the presence of SARS-CoV-2 in samples by RT-ddPCR (16).

**Figure 1:**
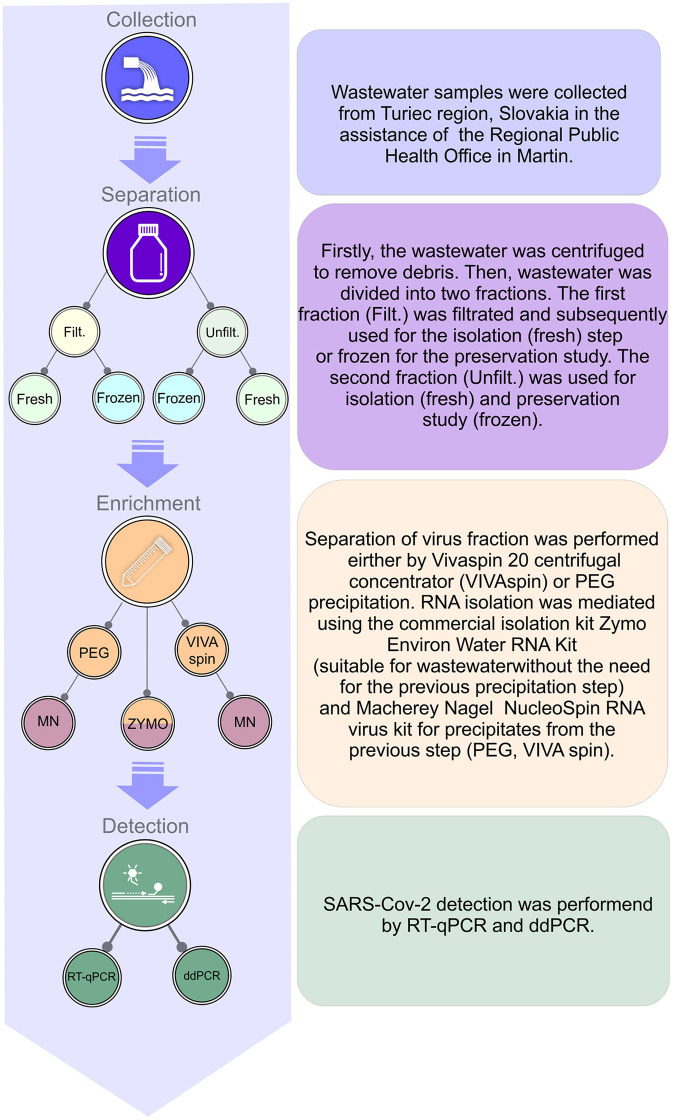
Schematic visualization of complete workflow for processing raw sewer sample, enrichment, isolation of RNA, and detection of SARS-CoV-2 in wastewater.

For the schematic visualization of the complete workflow, see Figure 1.

## 3. Results

### 3.1. Comparison of two detection methods for the detection of SARS-CoV-2 RNA/sensitivity

To perform the most precise detection of SARS-CoV-2 in wastewater, we compared two quantitative analysis methods, namely RT-qPCR (qTOWER—Analytic Jena) and ddPCR (QX200 Droplet digital PCR system—Biorad). All samples were analyzed in triplicates for higher statistical power of the experiment. The samples were collected three times from April to May 2022. Using filtered wastewater, RT-ddPCR identified 21 positive samples compared to 12 positive results analyzed by RT-qPCR. Furthermore, significant differences were detected in unfiltered wastewater in which RT-ddPCR identified 19 positive samples compared to 12 positive results detected by RT-qPCR (Table 1) Additional information is summarized in the Supplementary material (see Supplementary Table B in Supplementary material).

**Table 1:** Comparison of detection methods for viral RNA and workflows suitable for purification, concentration, and isolation of SARS-CoV-2 in wastewater.

**Filt./Unfilt. wastewater**	**Separation and isolation method**	**Sample (replicates)**	**Detection method (RT-ddPCR/RT-qPCR)**	**Wastewater collection**		
				**1**.	**2**.	**3**.
Filt.	PEG-8000	1	RT-ddPCR	+	+	+
		2		+	+	+
		3		+	+	?
		1	RT-qPCR	^*^	+	+
		2		^*^	^*^	^*^
		3		^*^	+	?
	Vivaspin	1	RT-ddPCR	+	+	?
		2		+	+	?
		3		?	?	–
		1	RT-qPCR	?	–	^*^
		2		?	–	–
		3		?	–	–
	Zymo	1	RT-ddPCR	+	+	+
		2		+	+	+
		3		+	+	+
		1	RT-qPCR	+	+	+
		2		+	+	+
		3		+	+	+
Unfil.	PEG-8000	1	RT-ddPCR	+	+	?
		2		+	+	+
		3		+	+	+
		1	RT-qPCR	?	^*^	?
		2		^*^	–	?
		3		–	+	?
	Vivaspin	1	RT-ddPCR	?	?	?
		2		+	^*^	–
		3		+	?	?
		1	RT-qPCR	?	^*^	^*^
		2		?	+	–
		3		?	+	–
	Zymo	1	RT-ddPCR	+	+	+
		2		+	+	+
		3		+	+	+
		1	RT-qPCR	+	+	+
		2		+	+	+
		3		+	+	+

RT-ddPCR, reverse transcription-droplet digital PCR; RT-qPCR, realtime-quantitative PCR; PEG-8000, polyethylene glycol-8000; Filt., filtered; Unfil., unfiltered.

+, positive; –, negative; ^*^, different coronavirus; ?, invalid result.

### 3.2. Comparison of three methods for the concentration and isolation of SARS-CoV-2 RNA

In our study, we compared three different methods for virus concentration and purification (Vivaspin and PEG) and RNA isolation, including NucleoSpin RNA Virus, Mini kit for viral RNA (Macherey-Nagel), and Zymo Environ Water RNA Kit (Zymo) to select the appropriate protocol for subsequent downstream analyses. Using PEG-8000 and NucleoSpin RNA Virus Mini kit, we identified eight positive results in filtered wastewater and nine positive results in unfiltered wastewater using RT-ddPCR. RT-qPCR detected three positive samples (in filtered wastewater), while only one sample was positive for SARS-CoV-2 in unfiltered wastewater. Virus separation using Vivaspin columns and subsequent isolation by NucleoSpin RNA Virus Mini kit detected SARS-CoV-2 positivity in four cases (filtered wastewater) compared to two positive outputs after RT-ddPCR analysis identified in unfiltered wastewater. The same workflow applied for RT-qPCR detected the absence of positivity in filtered wastewater; only two positive samples were confirmed in unfiltered wastewater. The protocol for virus purification and RNA isolation (Zymo Environ Water RNA Kit) identified positivity in all samples after RT-ddPCR as well as RT-qPCR analysis in both filtered and unfiltered wastewater (Table 1).

### 3.3. Analysis of the usage of unfiltered wastewater vs. wastewater filtered with 0.22 μm strainer using RT-ddPCR

**Figure 2:**
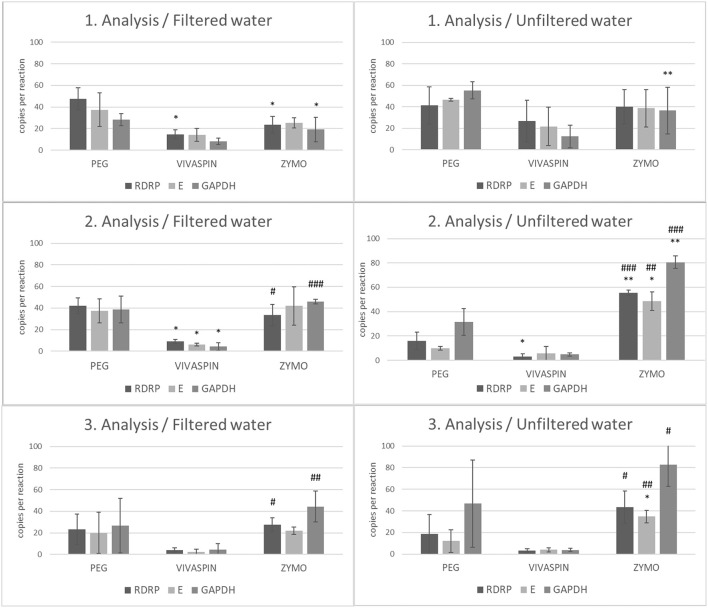
The number of copies of SARS-CoV-2 genes (*RdRp, E*) and *GAPDH* between filtrated and unfiltered wastewater processed by different separation and isolation protocols measured by RT-ddPCR. Acquired data are expressed as mean ± SEM. Significant difference, ^*^p < 0.05, ^**^p < 0.01 vs. PEG, ^#^*p* < 0.05, ^*##*^textitp < 0.01, ^*###*^*p* < 0.001 vs. VIVASPIN.

In accordance with our experiment workflow, the collected wastewater included: (1) filtrated with a 0.22 mm strainer or (2) used for further analysis without requiring a filtration step. In the next step, both wastewater samples (filtered/unfiltered) were processed by the aforementioned separation and isolation protocols (Table 1). Data were assessed as copies of viral RNA per reaction using RT-ddPCR. We observed a significantly lower number of copies of viral *RdRp* between samples processed by PEG and samples processed by Vivaspin columns and Zymo kit (*p* < 0.05) using filtered wastewater in the first analysis. Moreover, the level of *GAPDH* copies was significantly lower in samples isolated by Zymo kit compared to samples processed by PEG (*p* < 0.05). In unfiltered wastewater, the number of *GAPDH* copies was significantly lower (*p* < 0.01) in samples purified and isolated by Zymo kit than in samples after PEG precipitation and subsequent RNA isolation by the NucleoSpin RNA Virus kit. The second analysis of wastewater showed a significant decrease in the number of gene *E* (*p* < 0.05), *RdRp* (*p* < 0.05), and *GAPDH* (*p* < 0.05) copies in the samples concentrated by Vivaspin columns compared to samples after PEG precipitation and RNA isolation. Statistical significance was also observed in the number of *gene E* (*p* < 0.05), *RdRp* (*p* < 0.01), and *GAPDH* (*p* < 0.01) copies in samples processed by Zymo kit compared to samples after PEG-mediated virus precipitation and RNA isolation in unfiltered wastewater in the second round of analysis. In addition, a comparison between a number of gene copies in samples processed using Vivaspin and Zymo kit showed significant differences in *RdRp* (*p* < 0.05) and GAPDH (*p* < 0.01) (in filtered water) and *RdRp* (*p* < 0.001), *E* (*p* < 0.01), and *GAPDH* (p < 0.001) in unfiltered wastewater. The third analysis showed similar results between samples processed *via* Vivaspin and Zymo kit. The number of *RdRp* (*p* < 0.05) and *GAPDH* (*p* < 0.01) copies was significantly higher in filtered wastewater after the Zymo purification and isolation step. Also, there was statistical significance between *RdRp* (*p* < 0.05), *E* (*p* < 0.01), and *GAPDH* (*p* < 0.05) in unfiltered wastewater processed by Vivaspin and Zymo workflow. Finally, a significantly increased number of *RdRp* copies (*p* < 0.05) was observed in samples after Zymo processing than those after PEG separation in the third analysis. All data are summarized in Figure 2.

### 3.4. Analysis of the usage of unfiltered wastewater vs. wastewater filtered with 0.22 μm strainer using Zymo Environ Water RNA Kit analyzed by RT-ddPCR

**Figure 3:**
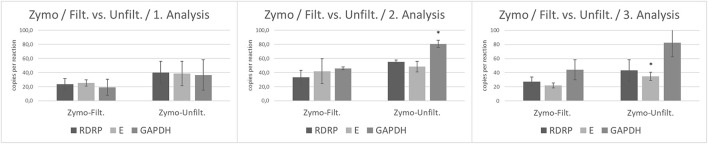
The number of copies of SARS-CoV-2 genes (*RdRp, E*) and *GAPDH* between filtrated and unfiltered wastewater processed by Zymo Environ Water RNA Kit analyzed by RT-ddPCR. Acquired data are expressed as mean ± SEM. Significant difference, ^*^p < 0.05 vs. PEG.

According to previous results, we compared unfiltered and filtered wastewater processed by Zymo Environ Water RNA kit. In the first analysis, there was no statistical significance between filtered and unfiltered wastewater in the number of viral gene copies (*E, RdRp*) as well as in housekeeping gene *GAPDH*. The second analysis evaluated by RT-ddPCR revealed differences between the amount of *GAPDH* copies (*p* < 0.05) in unfiltered wastewater compared to filtered wastewater. In the third analysis, we observed a significantly increased level of viral *gene E* (*p* < 0.05) in unfiltered wastewater (Figure 3).

### 3.5. Determination of suitability of the usage of frozen wastewater vs. fresh

**Figure 4:**
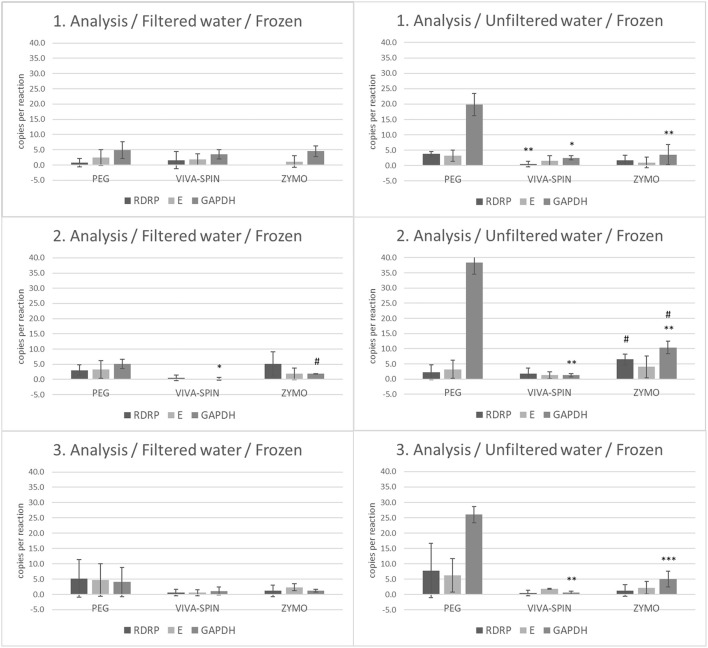
The number of copies of SARS-CoV-2 genes (*RdRp, E*) and *GAPDH* between frozen filtrated and unfiltered wastewater processed by different separation and isolation protocols measured by RT-ddPCR. Acquired data are expressed as mean ± SEM. Significant difference, ^*^p < 0.05, ^**^p < 0.01, ^***^vs. PEG,^#^*p* < 0.05 vs. VIVASPIN.

To determine the impact of thaw/freeze on the stability of viral RNA in wastewater samples, we analyzed frozen filtered and unfiltered wastewater (frozen aliquots from the first three collections). In the filtered fraction of wastewater, we observed a significantly decreased number of *GAPDH* copies in samples processed by Vivaspin protocol compared to PEG processing (*p* < 0.05) and an increased number of *GAPDH* in wastewater processed by Zymo kit compared to Vivaspin (*p* < 0.05) in the second analysis. On the other hand, unfiltered fractions manifested more diverse results. In the first analysis, the level of *GAPDH* was decreased in samples processed by both Vivaspin (*p* < 0.05) and Zymo kit (*p* < 0.01) protocols compared to PEG workflow. Moreover, the level of *RdRp* was lower (*p* < 0.01) in samples concentrated by Vivaspin columns than in those processed by PEG precipitation. The second analysis revealed a decrease in *GAPDH* (*p* < 0.01) in samples after Vivaspin centrifugation and Zymo kit processing compared to samples after PEG-mediated precipitation. Furthermore, significance was confirmed between a number of *RdRp* (*p* < 0.05) and *GAPDH* (*p* < 0.05) copies after purification and RNA isolation mediated by Zymo kit and samples selected for Vivaspin centrifugation. The last analysis of unfiltered frozen wastewater showed a significantly decreased level of *GAPDH* in samples after processing by Vivaspin (*p* < 0.01) and Zymo kit (*p* < 0.001) workflow than in samples processed by PEG precipitation (Figure 4).

### 3.6. Example of the usage of the selected protocol in practical settings

We received total 10 samples (analyzed as triplicates) of wastewater collected over the 2-month period between May 2022 and July 2022. Except for one replicate, all 10 samples were positive for SARS-CoV-2, analyzed by RT-ddPCR. On the other hand, results from RT-qPCR showed inconsistency characterized by negative and invalid results or the presence of different coronavirus (Table 2).

**Table 2:** Comparison of detection methods for viral RNA in weekly analyses of SARS-CoV-2 in wastewater.

**Detection method**	**Replicate**	**2.5**.	**9.5**.	**16.5**.	**23.5**.	**30.5**.	**6.6**.	**13.6**.	**20.6**.	**27.6**.	**4.7**.
		**2022**	**2022**	**2022**	**2022**	**2022**	**2022**	**2022**	**2022**	**2022**	**2022**
RT-ddPCR	1	+	+	+	+	+	+	+	+	+	+
	2	+	+	+	+	+	+	+	+	+	+
	3	+	+	+	+	+	–	+	+	+	+
RT-qPCR	1	+	+	–	+	^*^	?	–	^*^	+	+
	2	+	+	+	–	–	?	–	+	–	^*^
	3	+	+	–	+	–	–	+	+	+	^*^

RT-ddPCR, reverse transcription-droplet digital PCR; RT-qPCR, realtime-quantitative PCR.

+, positive; –, negative; ^*^, different coronavirus; ?, invalid result.

**Figure 5:**
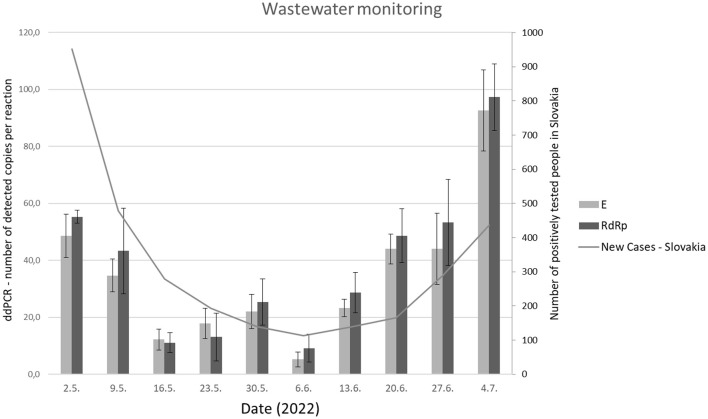
The number of copies of viral genes (*RdRp* and *E*) evaluated by RT-ddPCR periodically, from May 2022 to July 2022 in comparison with the epidemiology situation (new cases) in the Slovak republic.

Long-term monitoring revealed an increased amount of SARS-CoV-2 viral gene copies in wastewater samples compared to expectations based on national data acquired by individual testing using RT-qPCR (17). This trend was consistent throughout the last 5 weeks of the analysis (Figure 5).

## 4. Discussion

Waste-based epidemiology (WBE) of SARS-CoV-2 provides a powerful tool for epidemiological monitoring. Specifically, WBE analyses the signals of viral load in wastewater samples pooled by the whole population, regardless of symptoms occurrence, willingness to undergo the testing procedure, or to report the results to the authorities. Hence, WBE offers the possibility of an early warning system for the COVID-19 in the population. Therefore, WBE has rightfully became one of the most potent means for health authorities worldwide to monitor COVID-19 (18). In fact, the predictions of viral transmission dynamics based on such data are resistant to changes in the behavior of the public (e.g., testing practices, healthcare-seeking behavior, etc.) (19). On the other hand, certain limitations like environmental conditions (e.g., actual water temperature, dilution of wastewater due to the increased rainfall, the intermittent presence of chemical compounds that can act as PCR inhibitors, and sampling design) can potentially affect the results of WBE (12). Also, the access of particular demographic groups with specific, often risky, patterns of behavior to the sewage system can be limited. Despite these facts, which indeed need to be taken into consideration, WBE provides the most representative data source for epidemiological surveillance.

The importance of early SARS-CoV-2 detection in wastewater was documented in a recent study by Medema et al. The authors had observed the presence of SARS-CoV-2 RNA in sewage 6 days before the first cases reported in Amersfoort, Netherlands (20). Moreover, Randazzo et al. detected SARS-CoV-2 RNA in wastewater before the first COVID-19 cases confirmed by local authorities in the region of Murcia, Spain (10). Thus, the early identification of SARS-CoV-2 from sewage can play a crucial role in the surveillance of SARS-CoV-2 variants to support public health decision-making concerning measures to limit SARS-CoV-2 spread or allocation of testing or SARS-CoV-2 vaccination (21). Therefore, there is an imminent need to choose the most sensitive and cost-effective workflow for daily routine diagnosis of SARS-CoV-2 from wastewater as a tool to track COVID-19 incidence dynamics through time, even if the positivity rates tested by individual RT-qPCR or rapid antigen tests are low.

In addition to untreated wastewater, primary sludge can also be used as a primary source of viral RNA in the monitoring of the initial, exponential, and re-emergence phase at the epidemic level (22–24). Recent evidence proposed that using a wastewater sludge can be source of SARS-CoV-2 (enveloped virus in wastewater absorbed onto organic matter, resulting in a higher concentration of viral RNA in sludge) (25). Still, wastewater testing remains the most used approach for tracking COVID epidemiology that is appropriate for long-term monitoring of SARS-CoV-2 spreading on the regional level due to inexpensive and easy set-up for laboratory staff (26). However, using the sludge fraction is incompatible with our downstream protocol steps.

The majority of protocols for SARS-CoV-2 RNA isolation from wastewater use initial centrifugation for the removal of debris prior to processing (7, 27, 28). This step is important for reducing the turbidity of wastewater *via* removing larger particles and finer particles, which could inhibit PCR reaction as well as improve virus recovery (mainly for samples nearer the limit of detection) (7, 29–31).

In our study, we compared three different isolation and two detection methods. Centrifugal concentration through the Vivaspin column failed in our experimental settings, although such protocols were successfully performed by other groups (6, 29, 32). We can speculate whether it was caused by the specific physicochemical properties of local wastewater or by the presence of inhibitors that were not removed by filtering procedure, among others. However, due to limited capacity, we did not further investigate the failure of Vivaspin column, just concluded this method as not suitable for our conditions.

We are aware of the existence of multiple variants of PEG precipitation protocols including different combinations of usage of PEG-6000 (33, 34) or PEG-8000 (35, 36), different PEG concentrations ranging from 8%, 10% up to 50% (29, 34), different NaCl concentrations (37–39), and different times of incubation varying from 15 min to overnight (18 h) (11, 40). Not having the capacity to test all of them, we decided to use the protocol utilizing 10% PEG-8000 with 0.3 M NaCl overnight incubation. Despite hands-on experience with this particular procedure, which we performed successfully multiple times, we do not dare to claim it as an optimal technique. Our data are in agreement with Flood et al. and Câmara et al. who concluded that utilization of PEG method provide better virus recovery than the ultrafiltration-based methods (39, 41).

Nevertheless, we demonstrated that the best results were obtained with the Zymo Environ Water RNA Extraction Kit, which is dedicated for the isolation of the RNA from the water medium. Moreover, the Zymo Environ Water RNA kit was the most effective and efficient kit of the four commercial kits tested by O'Brien (42).

RT-qPCR is a commonly used mean of SARS-CoV-2 genome detection for both individual testing (13) and WBE. Only a minority of research groups have carried out molecular assays using RT-ddPCR (43). In contrast, RT-ddPCR demonstrated better results in detecting SARS-CoV-2 gene targets when compared with RT-qPCR in tested wastewater samples (39). According to our experience, RT-ddPCR could identify positive samples more reliably compared to RT-qPCR. For example, sampling from June 6, 2022 provided wastewater with contaminant causing darkish to the black coloration of the specimen that could not been removed by centrifugation nor filtration. The results of that day were particularly wrong (two positive results and one negative for RT-ddPCR vs. two invalid results and one negative for RT-qPCR) but still in a favor of RT-ddPCR. An explanation of this observation can be associated with the fact that ddPCR shows increased tolerance to inhibit substances due to the distribution and separation of individual micro-reactions, which mitigates the impact of inhibitors on PCR amplification by retaining discernible positive signal even if moderate PCR inhibition is occurring in a droplet (44). Moreover, ddPCR is considered to be more sensitive than RT-qPCR (45). These phenomena support the role of ddPCR as an attractive alternative to qPCR for diagnostic applications in conditions when increased sensitivity and processivity is necessary.

We have tested whether our protocol setup for SARS-CoV-2 isolation and detection would be functional if frozen samples were processed. The outcome clearly suggested that, even though the possibility of utilization of such stored material would be beneficial, our optimized workflow does not provide satisfactory results in this case.

Similarly, pre-treatment of wastewater by filtration through a 0.45 μm filter was not beneficial when Zymo Environ Water RNA Kit was used. On the contrary, several non-significant trends were observed in the case of Vivaspin ultrafiltration and PEG precipitation; however, we did not further investigate these two methods due to their inefficiency.

In this work, we have optimized protocol for the detection of SARS-CoV-2 RNA in wastewater in the conditions of our region. However, this workflow or its modifications can be utilized in similar environments elsewhere or can serve as a basis for the development of tools for WBE of SARS-CoV-2 or other pathogens that can be found in sewage system.

## 5. Conclusion

In conclusion:

◦ Usage of Zymo Environ Water RNA Kit provided superior quality of isolated RNA in comparison with both ultracentrifugation and PEG precipitation.
◦ RT-ddPCR outperforms RT-qPCR.
◦ Freezing of wastewater samples significantly reduces the RNA yield.
◦ Filtering is counterproductive when Zymo Environ Water RNA Kit is used.
◦ WBE is a useful and cost-effective tool for SARS-CoV-2 pandemic management with great potential for application on other pathogens.
◦ We have shown that the sensitivity of testing the samples with a nearer detection limit can be improved by selecting the appropriate combination of enrichment, isolation, and detection methods.

## Data availability statement

The raw data supporting the conclusions of this article will be made available by the authors, without undue reservation.

## Author contributions

VL and MS: conceptualization, investigation, formal analysis, methodology, validation, writing—original draft, review, and editing. TB, EL, ZK, VH, ET, and AH: methodology, validation, and writing—review and editing. TZ: review and editing, methodology, and resources. PP: investigation, methodology, review, and editing. LR: resources, methodology, review, and editing. ZD, EN, RP, AC, and EH: resources, writing—review and editing, and funding acquisition. All authors contributed to the article and approved the submitted version.
