# Supplementary material for: Comparison of the methods for isolation and detection of SARS-CoV-2 RNA in municipal wastewater
Source: Front Public Health. 2023 Mar 7;11:1116636. doi: 10.3389/fpubh.2023.1116636 (PMC10028190; doi:10.3389/fpubh.2023.1116636)
Supplement: Supplementary file 1 [file Data_Sheet_1.PDF]

Suppl. Table A. Comparison of the number of newly identified Covid patients on a national and regional scale.

	Date	Slovakia	Turiec region
**New SARS-CoV-2 cases, 7-day average**	2.5.	952	18
	9.5.	478	11
	16.5.	279	6
	23.5.	193	5
	30.5.	139	2
	6.6.	113	2
	13.6.	138	2
	20.6.	166	3
	27.6.	289	2
	4.7.	437	5
**Population**		5,447,000	112,000

Suppl. Table B. Comparison of detection methods for viral RNA and workflows suitable for purification, concentration, and isolation of SARS-CoV-2 in wastewater. Data from ddPCR are expressed as number of copies per reaction, for RT-qPCR as Ct value.

**Filt./Unfilt. wastewater**	**Separation and isolation method**	**Sample (replicates)**	**Detection method**	**Wastewater collection**											
				**1.**				**2.**				**3.**			
				RdRp	***E***	***GAPDH***	***IC***	***RdRp***	***E***	***GAPDH***	***IC***	***RdRp***	***E***	***GAPDH***	***IC***
Filt.	PEG-8000	1	RT-ddPCR (Copies)	36	20	27.2	-	40	48	37	-	36	38	50	-
		2		50	50	23.2	-	36	38	27.2	-	26	22	30	-
		3		56	42	34.4	-	50	26	52	-	8.2	0	0	-
		1	RT-qPCR (Ct)	No Ct	37.41	-	36.15	39.66	35.77	-	35.57	38.4	38.21	-	39.52
		2		38.03	No Ct	-	39.11	No Ct	36.02	-	34.32	No Ct	38	-	36.87
		3		48.05	37.41	-	36.06	38.71	36.83	-	35.83	No Ct	40.83	-	No Ct
	Vivaspin	1	RT-ddPCR (Copies)	16	8.2	10.4	-	9	7.8	7.2	-	4.4	5.2	0	-
		2		18	20	9.4	-	7	5.8	5.6	-	2.2	0	2.4	-
		3		10,2	14	4.6	-	11	5.4	0	-	5.8	1.8	10.6	-
		1	RT-qPCR (Ct)	No Ct	No Ct	-	No Ct	No Ct	No Ct	-	38.15	No Ct	38.53	-	36.05
		2		No Ct	No Ct	-	No Ct	No Ct	No Ct	-	34.63	No Ct	No Ct	-	37.72
		3		No Ct	No Ct	-	No Ct	No Ct	No Ct	-	35.96	No Ct	No Ct	-	36.29
	Zymo	1	RT-ddPCR (Copies)	30	28	18.8	-	22	36	44	-	20	20	29.4	-
		2		15	20	8.2	-	40	28	46	-	30	26	58	-
		3		26	28	30.8	-	38	62	48	-	32	20	45.6	-
		1	RT-qPCR (Ct)	33.7	34.26	-	33.5	33.91	32.64	-	32.09	35.17	34.31	-	33.65
		2		34.62	34.46	-	33.42	32.81	32.47	-	31.44	35.67	34.75	-	34.37
		3		33.43	33.59	-	33.05	33.5	32.81	-	32.17	35.4	34.95	-	34.37
-Unfil.	PEG-8000	1	RT-ddPCR (Copies)	56	46	48	-	18	11	22.8	-	0	0	0	-
		2		46	48	54	-	9.8	10.2	44	-	20	20	72	-
		3		22	46	64	-	20	8	28	-	36	16	68	-
		1	RT-qPCR (Ct)	No Ct	No Ct	-	No Ct	No Ct	38.03	-	35.39	No Ct	No Ct	-	No Ct
		2		No Ct	39.08	-	39.16	No Ct	No Ct	-	36.16	No Ct	No Ct	-	No Ct
		3		No Ct	No Ct	-	38.46	39.43	36.24	-	35.93	No Ct	No Ct	-	No Ct
	Vivaspin	1	RT-ddPCR (Copies)	4,6	1,4	1.6	-	0	3.2	4.2	-	1.8	4.8	3.8	-
		2		36	34	22.6	-	4.6	12	6.4	-	5	1.8	5.2	-
		3		40	30	13.4	-	4	1.8	3.8	-	2.4	5.4	2.4	-
		1	RT-qPCR	No Ct	No Ct	-	No Ct	No Ct	37.9	-	34.63	No Ct	38.18	-	35.92
		2		No Ct	No Ct	-	No Ct	38.49	35.48	-	34.75	No Ct	No Ct	-	36.5

		3	(Ct)	No Ct	No Ct	-	No Ct	37.16	36.98	-	33.94	No Ct	No Ct	-	39.91
	Zymo	1	RT-ddPCR (Copies)	28	24	17.2	-	54	54	76	-	26	28	60	-
		2		34	34	32.8	-	54	40	86	-	52	38	90	-
		3		58	58	60	-	58	52	80	-	52	38	98	-
		1	RT-qPCR (Ct)	33.35	33.49	-	33.02	33.22	33.14	-	32.1	36.26	35.81	-	34.38
		2		33.98	34.74	-	33.48	34.05	33.1	-	32.25	35.9	34.74	-	34.83
		3		33.62	33.4	-	32.76	33.24	32.44	-	31.43	35.45	36.4	-	34.33

Abbreviations: RT-ddPCR, Reverse transcription-droplet digital PCR; RT-qPCR, realtime-quantitative PCR; PEG-8000, polyethylene glycol-8000; Filt., filtered; Unfil., unfiltered

Explanatory notes: green = positive; red = negative; blue = different coronavirus; yellow = invalid result.
